# Effects of drying conditions in low‐temperature microwave‐assisted drying on bioactive compounds and antioxidant activity of dehydrated bitter melon (*Momordica charantia* L.)

**DOI:** 10.1002/fsn3.1676

**Published:** 2020-06-05

**Authors:** Thi‐Van‐Linh Nguyen, Quoc‐Duy Nguyen, Phuoc‐Bao‐Duy Nguyen, Bich‐Lam Tran, Phong T. Huynh

**Affiliations:** ^1^ Ho Chi Minh University of Technology (HCMUT) Ho Chi Minh City Vietnam; ^2^ Vietnam National University Ho Chi Minh City Vietnam; ^3^ Faculty of Environmental and Food Engineering Nguyen Tat Thanh University Ho Chi Minh City Vietnam

**Keywords:** DPPH, FRAP, low‐temperature drying, microwave‐assisted drying, total phenolic content, vitamin C

## Abstract

Bitter melon (*Momordica charantia* L.) is a fruit that brings health benefits to consumers because the fruit is rich in bioactive compounds. In this work, a combination of low‐temperature convective drying and microwave radiation was used to dehydrate sliced bitter melon. One‐factor‐at‐a‐time design was performed to evaluate the influence of microwave power density (1.5, 3.0, 4.5 W/g), drying temperatures (20, 25, and 30°C), and air velocity (1.0, 1.2 and 1.4 m/s) on the change of moisture content, nutrient levels (vitamin C and total phenolics), and the antioxidant activities (DPPH and FRAP assays) of the bitter melon. The obtained results showed that all investigated factors affected the rate of moisture removal. Microwave power density output and air‐drying temperature strongly participated in the retention of nutrients. In this study, the drying process was driven by both heat and mass transfer processes, so the increase of air velocity prolonged the drying time causing more loss of nutrient levels and antioxidant activities. It was found that DPPH free radical scavenging ability directly correlated with total phenolic content, but the ferric‐reducing antioxidant power was related to the presence of reductants including phenolic compounds, vitamin C, and other phytochemicals in bitter melons. This work determined that microwave power density and the air‐drying temperature are the main two factors that should be used for further investigations.

## Graphical abstract

The effects of drying conditions in the convective low‐temperature microwave‐assisted drying of bitter melon were investigated. The results were used to develop an understanding of how the microwave power density, drying temperature, and air velocity affect the final quality of the dried fruit product.

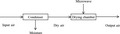

## 1 INTRODUCTION

Drying is an important operation in food processing to prolong the shelf‐life of perishable foods such as fruits and vegetables. Common drying methods such as hot air convection, vacuum‐drying, freeze‐drying, and infrared‐drying have been well studied and widely applied to dry these food commodities. Modern techniques, for example, microwave drying (MWD), microwave‐assisted drying (MWAD), or a combination of the microwave with traditional drying technology such as microwave vacuum‐drying or microwave freeze‐drying are highly interesting.

MWD or MWAD utilizes the advantage of a volumetric heating effect (Murthy & Prasad, 2005) to generate a co‐current gradient of relative humidity and temperature, leading to the acceleration of the moisture removal from the materials. It was reported that microwave drying of vegetables was improved or similar to conventional convective drying (Zhang, Tang, Mujumdar, & Wang, 2006). However, the major disadvantage of MWD or MWAD is its inconsistent heating, particularly at the edges of the sample, causing overheating (Nijhuis et al., 1998). To overcome the disadvantages of microwave drying, a combination of microwave and other drying techniques such as vacuum, freeze‐drying, or vacuum–freeze‐drying was recommended for fruit and vegetables. These combinations generated high‐quality dry products (Bondaruk, Markowski, & Błaszczak, 2007; Cui, Li, Song, & Song, 2008; Lin, Durance, & Scaman, 1998; Nahimana & Zhang, 2011; Xu, Zhang, Mujumdar, Duan, & Jin‐cai, 2006; Yanyang, Min, Mujumdar, Le‐qun, & Jin‐cai, 2004), but the cost of equipment and operation is relatively high (Gunasekaran, 1999; Yanyang et al., 2004). It would be economical if fruits and vegetables are dried at low temperature (less than or equal to 30°C) on microwave‐convective drying apparatus. The volumetric heating of microwaves enhances moisture diffusion, while huge air convection conveys both moisture and heat from the material.

Bitter melon (*Momordica charantia* L.) is a fruit rich of bitter substances which are highly biological activities. Bitter melon is a rich source of vitamins and phenolic compounds, which have been confirmed to bring health benefits including anticancer, antiviral, and anti‐inflammatory (Tan, Kha, Parks, & Roach, 2016). Natural antioxidants protect the human body from free radicals, and prevent oxidative stress and associated diseases (Barros, Cabrita, Boas, Carvalho, & Ferreira, 2011; Ferreira, Barros, & Abreu, 2009; López et al., 2007). It was found that the antioxidant capacity in fruits and vegetables (Kamiloglu et al., 2016) decreased due to chemical, enzymatic, or thermal decomposition of bioactive compounds (Nicoli, Anese, & Parpinel, 1999) during food processing. The vitamin C and phenolic compounds in bitter melon are prone to degradation during drying. Therefore, those compounds could be used as indicators of nutrient quality in drying food.

This study aimed to investigate the effects of low‐temperature microwave‐assisted drying conditions on bioactive compounds and antioxidant capacity of dehydrated bitter melon (*Momordica charantia* L.). Important factors, including microwave power density, air‐drying temperature, and air velocity, were used to design experiments. The effectiveness in removing moisture and quality of the dried product were used to evaluate levels of influence in each factor.

## 2 MATERIALS AND METHODS

### 2.1 Materials

**Figure 1:**
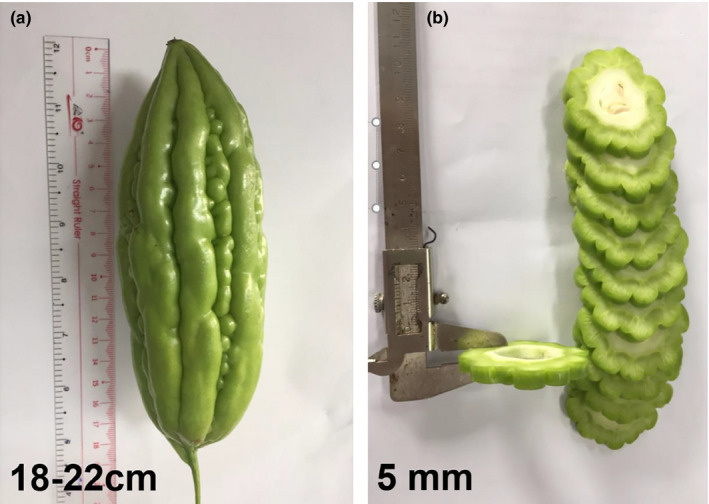
Fresh bitter melon fruit (a) and 5 mm thick sliced bitter melons (b)

Fresh bitter melons (*Momordica charantia* L.) were brought from the local markets in Ho Chi Minh City, Vietnam, based on ASEAN Standard ("ASEAN Stan^47^", 2016). High quality of bitter melons, which were from 18 to 22 cm in length and evenly green surface without visual defects, were selected. The bitter melons were washed, sliced into 5 mm thick, and deseeded (see Figure 1), and then, the central cylindrical part of the fruits was used to dry. The moisture content was determined using the oven method (AOAC, 2000).

Folin–Ciocalteu's reagent, gallic acid, and ascorbic acid (99.7% purity) were purchased from Sigma‐Aldrich. All the chemicals, including anhydrous 2,6‐dichlorophenolindophenol sodium salt (DCPIP), metaphosphoric acid, tripyridyltriazine, and sodium carbonate (99.5% purity) were used as received. Aqueous solutions were prepared using distilled water.

### 2.2 Microwave‐assisted drying equipment and drying conditions

**Figure 2:**
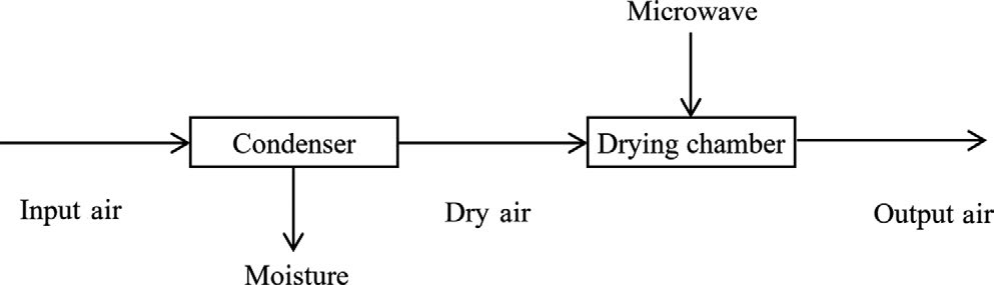
The principle of low‐temperature drying‐assisted microwave equipment

A custom‐built convective low‐temperature microwave‐assisted drying equipped with laboratory scale was used to dry the bitter melon slices. The principle of this drying method was performed in Figure 2. In this study, the magnetron had a maximum power output of 750 W at 2,450 MHz was used to generate microwave energy. The dry air used as a drying agent was obtained by removing moisture from the ambient moist air using a custom‐built dehumidifier (modified from a 2HP Toshiba refrigerator). Then, the dry air was passed through two electrical heaters to reach the required temperature before entering the drying chamber.

In this study, the one‐factor‐at‐a‐time method was applied to design experiments with three replications. Investigated factors include microwave power density (MWPD), which was defined as the amount of microwave power in watts per gram of drying material at the initial time, air‐drying temperature (*T*_air_), and air velocity. The levels of MWPD were 1.5, 3.0, and 4.5 W/g, respectively, those of *T*_air_ were 20, 25, and 30°C, correspondingly, and velocity of air (measured by a Tecpel digital mini anemometer AVM 713) were 1.0, 1.2, and 1.4 m/s, individually. The samples were put the symmetrical, single layer on a glass stage (turnable), which was mounted in the drying chamber and rotated at 4 rpm to ensure uniform heating. The samples were dried in 10 min and took rest in 2 min for weighting. The drying process was stopped when the moisture content of the slices approached about 0.1 g water/g d.b because it was reported that this value ensured the microbiological stability of the product (Ghanem, Mihoubi, Kechaou, & Mihoubi, 2012). Then, an aliquot of the dried bitter melon slices was chemically analyzed the contents of vitamin C, TPC, and antioxidant capacity (DPPH and FRAP assays).

### 2.3 Determination of pH

pH was determined using HI 2211–02 pH meter (Hanna Instruments).

### 2.4 Determination of Total phenolic contents

A precisely weighed aliquot (2.0 g) was crushed and ground with a few milliliters of distilled water by pestle and mortal at the ambient temperature. The aqueous extract was endured vacuum filter through a Whatman No. 1 filter paper and washed with distilled water until an aliquot of the extract showed no color change upon adding a few drops of the Folin–Ciocalteu reagent. The extract was taken in a 50‐ml volumetric flask and made up to the calibration mark by distilled water.

The TPCs were determined based on the Folin–Ciocalteu assay, using gallic acid as standard (Singleton, Orthofer, & Lamuela‐Raventós, 1999). 1 ml of Folin–Ciocalteu's reagent (diluted by distilled water to a concentration of 0.2 N) and 1 ml of sodium carbonate (20% w/v) were added to 1 ml of the diluted sample extract; then, the mixture was thoroughly mixed. The sample was placed in the dark for 30 min before being measuring the absorbance at 765 nm using a Shimadzu UV‐1800 spectrophotometer (Shimadzu Inc.). TPCs were expressed as gallic acid equivalent (GAE) in mg/100 g (dry basis).

### 2.5 Determination of total reducing sugar

Total reducing sugar contents were analyzed using the dinitrosalicylic acid (DNS) method (Miller, 1959). The DNS reagent was prepared by dissolving 1 g solid DNS in 100 ml of 0.4 M NaOH solution. Subsequently, 30 g of solid sodium potassium tartrate was added to yield the reagent. Samples were extracted using a procedure similar to that used for TPC determination (cf. Section 2.4). 2 ml of each sample was transferred to a glass test tube subsequent to the addition of 1 ml DNS reagent. After boiling for 10 min, the test tubes were cooled to the ambient temperature, and the absorbances were recorded at 540 nm. The results were expressed as glucose equivalent in g/100 g (dry basis).

### 2.6 Determination of Vitamin C contents

Vitamin C from the bitter melon samples was extracted using a procedure similar to that used for TPC determination (cf. Section 2.4), but the aqueous metaphosphoric acid (3% w/v) was used as the extraction solvent instead of distilled water. The vitamin C content of dried samples was determined according to AOAC’s official titrimetric method (AOAC Method 967.21) (Nielsen, 2017). Briefly, 2,6‐di‐chloroindophenol (DCPIP), a blue dye, generates a distinguished rose‐pink color in aqueous acidic solution. It forms a colorless solution when reacting with ascorbic acid. Thus, the adding of ascorbic acid into an acidic aqueous DCPIP solution drives its rose‐pink color faded continuously into colorless. The DCPIP solution was standardized using a standard solution of ascorbic acid before titration. A 5‐mL aliquot of the metaphosphoric acid extract of the sample was titrated against the standardized DCPIP solution above. The endpoint was detected when excess DCPIP gave a light but distinct rose‐pink color that persisted for more than 10 s. Vitamin C content was expressed in mg/100 g (dry basis) and calculated using the following equation: $VitC=\left(V_{s}-V_{0}\right)\times \frac{F}{m}\times \frac{V}{V_{t}}\times 100$ , where *V_s_* is the average volume for sample titration, *V_o_* is the average volume for blank titration, *F* is mg ascorbic acid equivalent to 1.0 ml DCPIP standard solution, *m* is solid sample weight, *V* is diluted volume, and *V_t_* is the volume of aliquot titrated.

### 2.7 The 2, 2‐diphenyl‐l‐picrylhydrazyl assay (DPPH)

The DPPH assay determination was modified from the method by Braca et al., (2001). Briefly, samples were extracted using a procedure similar to that used for TPC determination (cf. Section 2.4) but using methanol as the extraction solvent instead of distilled water. The extracts were transferred to a 100‐mL volumetric flask, diluted to the calibration mark by methanol. 0.2 ml of the extract was mixed with 3 ml DPPH solution (diluted in methanol to a concentration of 10^–4^ M) followed by shaking and incubating within 30 min in the dark before the absorbance measurement at 515 nm. The results were expressed as Trolox equivalent (TE) in mg/100 g (dry basis).

### 2.8 Determination of ferric‐reducing antioxidant power (FRAP)

The extract for DPPH determination above (cf. Section 2.7) was also used to characterize the ferric‐reducing antioxidant power (FRAP) assay by modifying the method proposed by Braca et al. (2001). First of all, FRAP reagent was prepared by mixing 0.3 M acetate buffer (pH 3.6), 0.01 M tripyridyltriazine solution (prepared in 0.04 M HCl), and 0.02 M FeCl3 solution at volumetric ratio of 10:1:1, respectively. 0.2 ml of the extract was mixed with 3 ml FRAP solution. The mixture was shaken and incubated for 30 min in the dark, and then quantified after recording the absorbance at 593 nm. The results were expressed as Trolox equivalent (TE) in mg/100 g (dry basis).

### 2.9 Statistical analysis

All experiments were conducted in triplicate. The mean and standard deviation of the mean were calculated in WPS Spreadsheets Software (Microsoft Inc.). Significant differences at *p* < .05 level of data were tested by Tukey's test.

## 3 RESULTS AND DISCUSSION

### 3.1 Physicochemical properties of fresh bitter melon

Fresh bitter melon was chosen from the local market in Vietnam following ASEAN Stan 47:2016 for representative samples. The bitter melons were analyzed their physicochemical properties, which were shown in Table 1. The bitter melon used is low‐acid fruit. The amount of total phenolics in the samples is much higher than that in broccoli (6.7 mg/g d.b.) (Koh, Wimalasiri, Chassy, & Mitchell, 2009). Vitamin C content in the samples is close to that in strawberry (5.9 mg/g d.b.) ("Strawberry & raw", 2018). The reducing sugar content in the fruit is also very low. Low reducing sugar content brings advantage in drying since reducing sugar participates in the Maillard reaction, which not only changes the color of the dry products but also involves in the phenolics degradation reaction (Tan & Harris, 1995).

**Table 1:** Characteristics of fresh bitter melon used^a^

Initial moisture content (%)	13.9 (0.5)
Phenolic compound conc. (mg/g)	8.0 (0.4)
Vitamin C conc. (mg/g)	5.2 (0.4)
Reducing Sugar conc. (mg/g)^b^	36 (7)
DPPH free scavenging ability (mg/g)	14.27 (0.43)
Ferric‐reducing power ability (mg/g)	53.13 (1.75)
pH^c^	5.4 (0.1)

^a^ Data are expressed as mean value (standard deviation), and chemical concentrations are based on dry mass.

### 3.2 Effect of microwave powers density output

**Figure 3:**
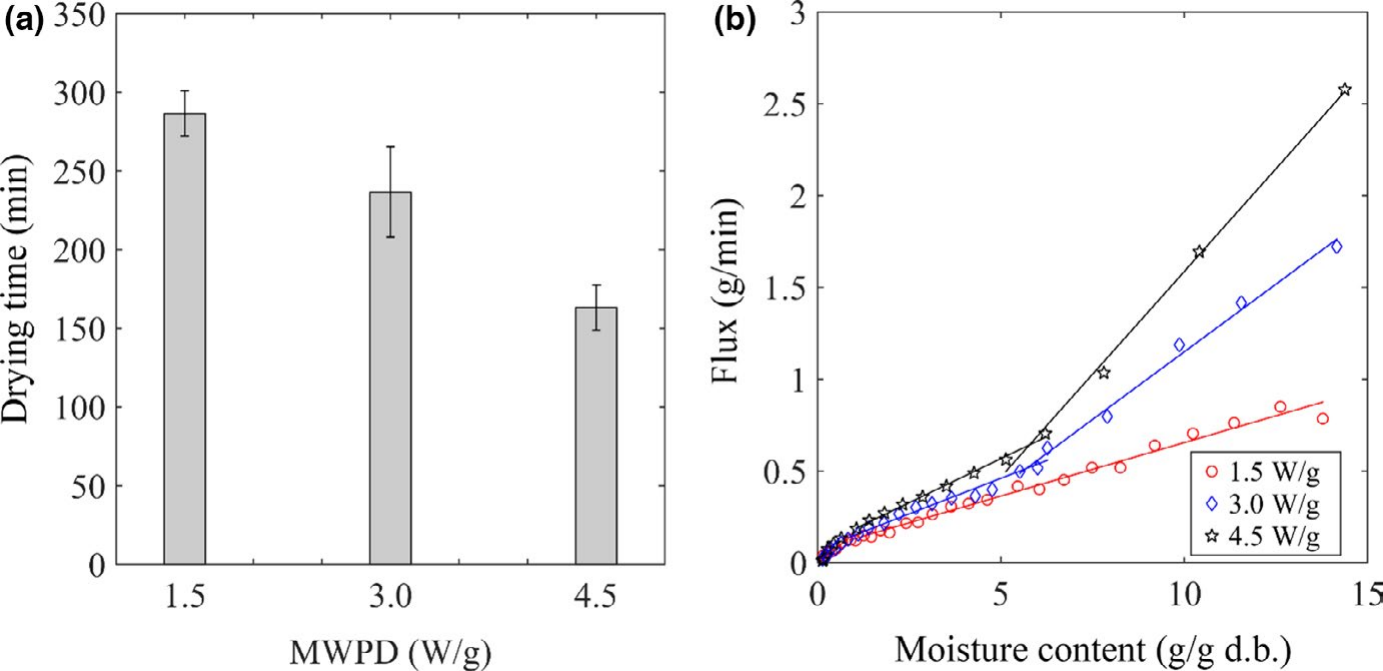
Impact of MWPD on the drying of bitter melon. (a) Drying time at different MWPD outputs. (b) Evaporation flux from initial moisture content to final moisture content of 0.1 g/g d.b

The effect of MWPD output on the drying of the bitter melon was investigated at the air velocity rate of 1.0 m/s and air temperature of 30°C. The results indicated that MWPD output significantly impacted the moisture removal from the samples (see Figure 3a). The time to dry the bitter melons (see Figure 3a.) reduced around 18% when MWPD output increased double (from 1.5 to 3.0 W/g). The reduction in drying time became 39% when the triple amount of MWPD output (4.5 W/g) was applied. Water is the main component which adsorbs and converts energy from microwave radiation to thermal energy used both to vaporize the moisture and heat the materials. The vaporization of water inside the samples creates a diffusivity driving force to push moisture from inside to the air–solid interface. Thus, the higher the MWPD output was, the more energy was absorbed by the samples leading to accelerating the sample drying periods. It was also reported that the drying time decreased significantly when the high level of MW power was applied in microwave vacuum‐drying of shiitake mushroom (Kantrong, Tansakul, & Mittal, 2014) or microwave‐assisted drying of other vegetables such as mushroom (Giri & Prasad, 2007), banana (Pereira, Marsaioli, & Ahrné, 2007), and pineapple (Botha, Oliveira, & Ahrné, 2012).

The flux of evaporation was estimated as $f\left(g/s\right)=\frac{\mathrm{dm}}{\mathrm{dt}}$ ; here, *dm* is the difference in mass of the sample corresponding to dt, which is the interval of drying time. Evaporation flux increased with the increase of MWPD (see Figure 3b). When high MWPD output, that is, 3.0 or 4.5 W/g, was applied, the flux of evaporation consisted of three distinguish parts separated by their slope. The first stage started at the highest flux of evaporation and steeply decreased until the moisture content in the sample approached its concentration of 5.5 g/g d.b. The slope of evaporation flux in the second part was not as steep as that of the first part. At the end of the drying, the slope of evaporation flux increased again when the moisture content in the sample reached 0.4 g/g d.b (approximately 28.57% of total water). Since then, there was no difference in evaporation flux among levels of MWPD output. When low MWPD output was used, the distinction between the slope of evaporation flux in the first and the second part became unclear, or undetectable. Water was found to allocate at three different compartments, which were vacuole, cytoplasm, and cell wall/extracellular space in plant tissues (Snaar & Van As, 1992). It was reported that free water (around 95%) mainly allocated in the vacuole and the xylem (Xu, Jin, Zhang, & Chen, 2017). The rest was bond water that consisted of approximately 3% of water‐binding with macromolecules in the cytoplasm, and around 2% water strongly interacting with cell wall/extracellular space (Xu et al., 2017). In our situation, the last stage of drying associated with the removal of bond water, which strongly interacted with macromolecules in the cytoplasm and cell wall. When high MWPD output irradiation was applied, the first stage of drying related to the evaporation of free water in vacuole and xylem, the second one associated with the rest of free water and portion of the water bond with macromolecules such as protein in the cytoplasm.

Results of vitamin C contents, TPCs, and antioxidant capacities (DPPH and FRAP assays) in dried samples at different MWPD were exhibited in Table 2. In the previous study, both vitamin C and TPC in bitter melon slices were susceptible to enzymatic degradation during the low‐temperature microwave‐assisted drying (Nguyen et al., 2019). Therefore, the content of bioactive compounds was reduced significantly after the drying process. Interestingly, the concentrations of both vitamin C and TPC were the highest when the bitter melon was dried at MWPD output of 3.0 W/g. Energy radiation from MW had the power to weaken polar bonds in a molecule, such as vitamin C and polyphenol compounds that have several polar bonds. Those bonds vibrate dramatically under microwave irradiation. That caused the bond rupture to induce chemical reaction (Dudley, Richert, & Stiegman, 2015) and enhanced catalytic reaction, including enzymatic reaction (Young, Nichols, Kelly, & Deiters, 2008). Therefore, the higher the MWPD output was, the faster the degradation reaction was. Another influence of MW on the degradation of the bioactive compounds in the bitter melons is reaction time and also the drying time. The longer reaction time is, the larger amount of bioactive compounds lose. Thus, it is not surprised at all to observe the retention of both vitamin C and phenolics in the samples dried at MWPD output of 1.5 W/g and 4.5 W/g was smaller than those at MWPD output of 3.0 W/g. The drying time and also the reaction time at MWPD of 1.5 W/g is 280 min. Therefore, long reaction durations played an important role at low MWPD drying. On the other hand, the influence of microwave on reaction rate dominated at high MWPD (4.5 W/g).

**Table 2:** Vitamin C and phenolic contents and antioxidant activities of dried bitter melon samples (the DPPH and FRAP assay) at different microwave power

MWPD (W/g)	Vitamin C content (mg/100g d.b.)	TPC (mgGAE/100g d.b.)	Free radical scavenging ability (DPPH assay) (mgTE/100g d.b.)	The ferric‐reducing ability (FRAP assay) (mgTE/100g d.b.)
1.5	143.24 (6.36) ^a^	359.46 (13.98) ^a^	144.32 (7.64) ^a^	1,384.67 (19.22) ^a^
3.0	161.15 (4.16) ^b^	459.04 (15.22) ^b^	215.32 (5.05) ^b^	1759.93 (42.15) ^b^
4.5	125.63 (7.80) ^c^	427.51 (11.77) ^c^	196.51 (8.33) ^c^	1,308.57 (32.10) ^a^

Note

Data are expressed as mean (standard deviation) and values within a column with the different letter are significantly different (*p* < .05).

Bitter melon is a beneficial health food because of its physiological function of antioxidation, antimicrobials, and cancer resistance (Othman, Ismail, Ghani, & Adenan, 2007). Biological compounds are found in raw materials such as vitamins and phenolic compounds. These natural compounds play an important role in contributing to the antioxidant activity of the material (Rice‐Evans, Miller, & Paganga, 1997). Phenolic compounds, as well as vitamins C and E, possess the ideal structure to act as free radical reducing agents and inhibit the formation of free radicals under the catalysis of transition metals. Thus, radical scavenging and metal ion reducing capacity have been used to evaluate the antioxidant capacity of vitamins (Nimse & Pal, 2015) and phenolic compounds (Huyut, Beydemir, & Gülçin, 2017; Loganayaki, Siddhuraju, & Manian, 2013). At least two methods should be employed to evaluate the total antioxidant activity due to different mechanisms of antioxidants, such as prevention of chain initiation, decomposition of peroxides, prevention of continued hydrogen abstraction, free radical scavenging, reducing capacity, and binding of transition metal ion catalysts (Inchuen, Narkrugsa, & Pornchaloempong, 2010). Therefore, DPPH and FRAP assays were used to determine the antioxidant activity of the samples under various drying conditions (Mao, Pan, Que, & Fang, 2006). As shown in Table 2, the free radical scavenging activity and FRAP power, which are highly associated with the vitamin C and TPC, of dried samples were the highest at MWPD output of 3.0 W/g.

### 3.3 Effect of the air‐drying temperature

**Figure 4:**
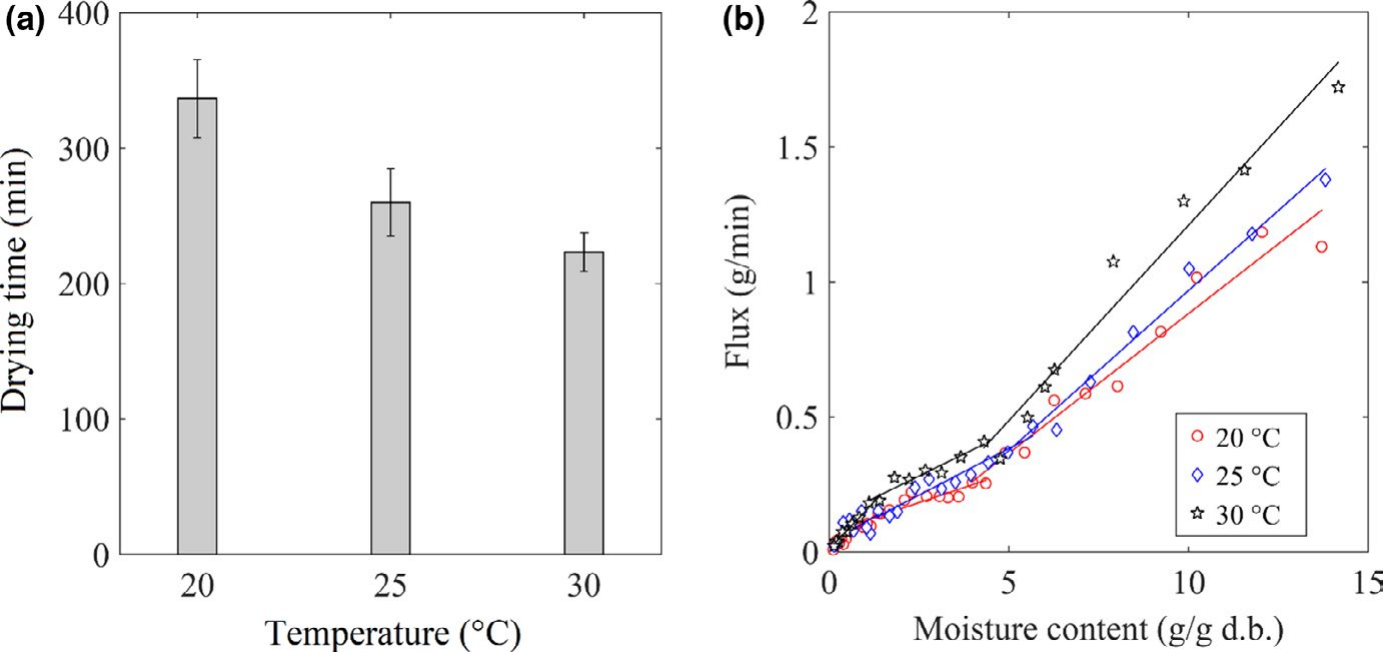
Impact of the air temperature on the MWAD. (a) Drying time at the different Tairs. (b). Evaporation flux at different temperatures of drying air

The effect of *T*_air_ on the drying of the sample was carried at the air velocity rate of 1.0 m/s and MWPD output of 3.0 W/g. Figure 4a shows the evaporation flux at different *T*_air_. The results showed that the drying time was 320, 260, and 230 min at 20, 25, and 30°C, respectively. At the same absolute humidity, the higher *T*_air_ was, the lower relative humidity was, thus increased the moisture evaporation rate at the material surface, leading to reduced drying time. Some studies on microwave drying under the influence of drying temperature such as blueberries (Zielinska & Michalska, 2016) and soybean (Gowen, Abu‐Ghannam, Frias, & Oliveira, 2008) showed that the increase in the temperature would shorten the drying time. However, the advantages of the increase in *T*_air_ in drying may not overcome its disadvantages, such as net energy consumption and the nutrient retention of dried products.

Table 3 shows the change of vitamin C, TPCs, and antioxidant capacity at different *T*_air_ s. The results showed that the maximum content of vitamin C was found to be the highest in the samples dried at *T*_air_ of 30°C. However, all other criteria, such as TPCs, free radical scavenging, and ferric‐reducing ability, were the highest in the samples dried at *T*_air_ of 25°C. The retention of nutrients depended on not only the rate of moisture removal but also the degradation rate. The removal of moisture from the materials increased nutrient concentration. However, bioactive compounds like vitamin C and phenolic compounds are prone to be decomposed during food processing. The higher air‐drying temperature is the shorter drying time (see Figure 4b) and also the higher rate of degradation.

**Table 3:** Vitamin C and phenolic contents and antioxidant activities of dried bitter melon samples (the DPPH and FRAP assay) at different drying air temperature

Drying temperature (°C)	Vitamin C content (mg/100g d.b.)	TPC (mgGAE/100g d.b.)	Free radical scavenging ability (DPPH assay) (mgTE/100g d.b.)	The ferric‐reducing ability (FRAP assay) (mgTE/100g d.b.)
20	128.10 (14.85) ^a^	423.37 (27.64) ^a^	184.64 (5.40) ^a^	1679.19 (36.48) ^a^
25	148.65 (6.40) ^b^	492.44 (20.09) ^b^	235.79 (13.38) ^b^	1802.26 (41.78) ^b^
30	169.96 (7.23) ^c^	440.78 (27.85) ^a^	209.14 (9.63) ^c^	1791.90 (34.80) ^b^

Note

Data are expressed as mean (standard deviation) and values within a column with the different letter are significantly different (*p* < .05).

The highest DPPH radical scavenging and TPC values were also found in the samples dried at 25°C indicating that DPPH free radical scavenging was direct correlations to TPC. Correlations between TPCs and DPPH radical scavenging activity were also reported in other studies about the drying of fruits and vegetables (Inchuen et al., 2010; İzli, Yıldız, Ünal, Işık, & Uylaşer, 2014; Sultana, Anwar, Ashraf, & Saari, 2012). In this study, it was found that the DPPH radical scavenging ability and TPC had a positive correlation with high R‐squared value (0.94). However, the ferric‐reducing ability in dried samples increased when the air‐drying temperature increased, and there was an insignificant difference with FRAP power at 25°C and 30°C. Thus, ferric‐reducing ability was not always proportional to DPPH radical scavenging ability, such as in this case, the correlation coefficient between free radical scavenging and ferric‐reducing ability was low (*R* ^2^ < .80). It was found that the reducing power had a positive correlation with the total content of vitamin C and TPC. So, it could be due to the reducing power was related to the presence of reductants, including TPC, vitamin C, and other non‐nutrient phytochemicals in bitter melons. Our results highly agree with the finding reported in other publications about microwave drying of Enicostemma littorale Blume (Sathishkumar, Lakshmi, & Annamalai, 2009) and Sage (*Salvia officinalis* L.) plants (Hamrouni‐Sellami et al., 2013).

### 3.4 Effect of the air velocity

**Figure 5:**
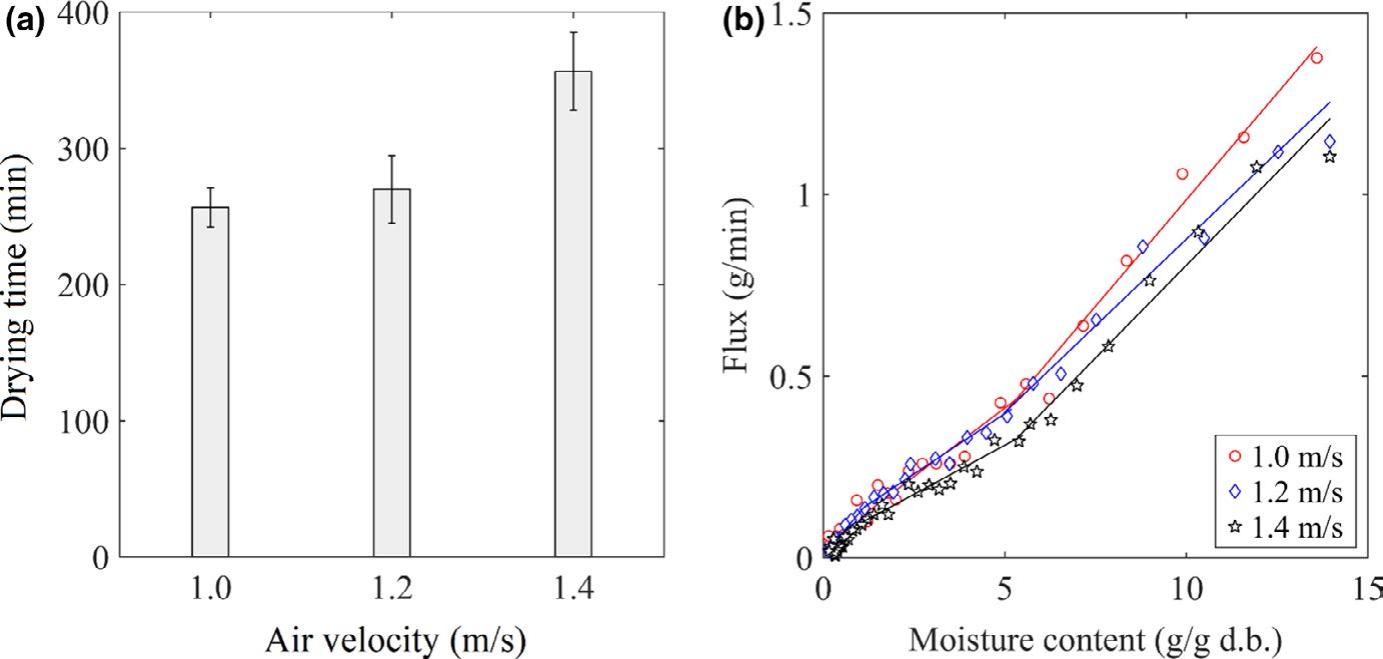
Impact of air velocity on the MWAD of bitter melon. (a) The drying time at different air velocities. (b) Evaporation flux at different air velocities

The biggest challenge of drying at low temperatures is the small driving force of evaporation because of high relative humidity in the air. Thus, increasing air velocity would be a good way to improve the evaporation flux since the velocity of the drying air correlates to convective mass transfer at the solid–air interface, that is, Schmidt and Sherwood number, $Sh=\frac{k_{c}L}{D_{12}}$ ; here, k_c_ is the convective mass transfer coefficient, L is characteristic length, and D_12_ is the diffusivity of water vapor in the air. To explore the impact of air velocity on the LTMWAD, the samples were dried at MWPD output of 3.0 W/g, *T*_air_ of 25°C, and the velocity of air ranged 1.0, 1.2, and 1.4 m/s. Reynolds number at the solid–air contacting phase ranged from 1503 to 2,119 based on characteristic length equal average differences between the external diameter and the internal diameter of bitter melon slices. It was implied that the mass transfer occurred in the laminar zone of the flow. The binary diffusion was estimated from the surface temperature of bitter melon slices using the Engineering Equation Solver database. Schmidt number of drying conditions was 0.74. The external forced convective mass transfer coefficients of the drying conditions correlated to the average Sherwood number for a flat plate $\bar{\mathrm{Sh}}=0.664{\text{Re}}^{\frac{1}{2}}Sc^{\frac{1}{3}}$ . Sherwood number indicates that the larger drying air velocity is, the greater evaporation flux is. Besides, when a process of heat and mass transfer occurs throughout convection, the fluid flow could be characterized using the Lewis number, $Le=\frac{\alpha }{D_{12}}$ ; here, α is thermal diffusivity. In this study, the values of the Lewis number were found 1.055, 1.055, and 1.056 for the air velocity of 1.0, 1.2, and 1.4 m/s, respectively. According to Hatami and Ganji (2014), if Lewis number equals 1, the operation is driven by both heat and mass transfer processes (Hatami & Ganji, 2014). It is not surprising that the large velocity of the drying air was, the longer the drying time was, and the slower evaporation flux was (see Figure 5) because the diffusion of water from the bulk of samples controlled the low temperature during drying. Therefore, the increase in air‐drying velocity does not reduce the drying time but the temperature of the samples. Similar observations were also reported in MWD of banana (Pereira et al., 2007) and garlic cloves (Sharma & Prasad, 2006).

Table 4 displays the change of nutrient contents (vitamin C and phenolic) and antioxidant capacity (the DPPH and FRAP assays) in dried samples at different air velocity. Results showed that the changes in the nutrient contents and antioxidant capacity in the samples dried at 1.0/s and 1.2 m/s were insignificant. However, those in the samples dried at an air velocity of 1.4 m/s significantly reduced because it accounted for the longer drying time at this condition. Long‐term exposure to microwave radiation made sensitive components (vitamin C and phenolic compounds) vulnerable to decompose, leading to the reduction in the antioxidant capacity.

**Table 4:** Vitamin C and phenolic contents and antioxidant activities of dried bitter melon samples (the DPPH and FRAP assay) at different air velocity

Air velocity (m/s)	Vitamin C content (mg/100g d.b.)	TPC (mgGAE/100g d.b.)	Free radical scavenging ability (DPPH assay) (mgTE/100g d.b.)	The ferric‐reducing ability (FRAP assay) (mgTE/100g d.b.)
1.0	147.00 (4.81) ^a^	494.60 (13.39) ^a^	230.85 (13.62) ^a^	1774.62 (46.16) ^a^
1.2	136.85 (8.54) ^a^	475.88 (8.53) ^a^	183.02 (12.57) ^a^	1664.46 (42.76) ^a^
1.4	108.14 (4.86) ^b^	456.38 (8.80) ^b^	141.91 (9.09) ^b^	1,322.40 (58.58) ^b^

Note

Data are expressed as mean (standard deviation) and values within a column with the different letter are significantly different (*p* < .05).

## 4 CONCLUSIONS

Low‐temperature (up to 30°C) convective microwave‐assisted drying is a new technique that can be used to dehydrate products containing heat‐sensitive components. However, until now, this potentially economic method has received little attention. The effect of drying conditions, including MWPD output, *T*_air_, and air velocity on drying of bitter melon as well as its nutrient content and antioxidant capacity, were investigated. The assistance of microwave irradiation showed effectiveness in removing moisture from the material at convective low‐temperature air‐drying. All of the investigated factors significantly affected moisture removal. MWPD output and *T*_air_ also showed significant effects on the retention of bioactive compounds and antioxidant capacity. However, high air velocity prolonged drying time, causing more loss in vitamin C, TPC, and antioxidant capacity. Changes in antioxidant capacity (DPPH assay) were found to be consistently correlated with changes in TPC. The results determine that MWPD output and *T*_air_ are the main factors that should be used for further investigation. Kinetics of moisture removal or nutrient degradation during drying will be the next consideration to provide more information on MWAD of materials rich in bioactive compounds. Besides, it is necessary to discover the impact of blanching treatment to inactivate enzymes that associate with the degradation of bioactive compounds.

## CONFLICT OF INTEREST

The authors declare no conflict of interest.

## AUTHORS’ CONTRIBUTIONS

Thi‐Van‐Linh Nguyen and Quoc‐Duy Nguyen contributed to investigation; Thi‐Van‐Linh Nguyen, Phong T. Huynh, and Bich‐Lam Tran contributed to methodology; Phuoc‐Bao‐Duy Nguyen contributed to data analysis; Thi‐Van‐Linh Nguyen contributed to writing—original draft; Phong T. Huynh contributed to review and editing.
